# Efficacy and safety of abdominal acupuncture in Parkinson’s disease: A protocol for systematic review and meta-analysis

**DOI:** 10.1097/MD.0000000000031804

**Published:** 2022-11-25

**Authors:** Yuehong Fang, Yilian Xu, Zhengzhong Liu, Sihan Dong, Ying Su

**Affiliations:** a School of Traditional Chinese Medicine, Changchun University of Chinese Medicine, Changchun, China; b School of Basic Medicine, Changchun University of Chinese Medicine, Changchun, China.

**Keywords:** abdominal acupuncture, Parkinson’s disease, protocol, systematic review

## Abstract

Parkinson disease (PD) is a worldwide spread neurodegenerative disorder. Dopamine replacement therapy is currently the mainstream treatment, which can alleviate the symptoms but induces motor complications. Acupuncture therapy is effective for PD. As a form of acupuncture, the abdominal acupuncture has been used to relieve symptoms in patients with PD, but its effectiveness and safety have not yet reached a definitive conclusion. Therefore, this systematic review and meta-analysis protocol is planned to evaluate the efficacy and safety of abdominal acupuncture for PD patients.

**Methods:**

Six English databases (PubMed, Web of Science, MEDLINE, EMBASE, Springer Cochrane Library, and WHO International Clinical Trials Registry Platform) and 4 Chinese databases (Wan Fang Database, Chinese Scientific Journal Database, China National Knowledge Infrastructure Database, and Chinese Biomedical Literature Database) will be searched normatively according to the rule of each database from the inception to August 20, 2022. Two reviewers will independently conduct article selection, data collection, and risk of bias evaluation. Any disagreement will be resolved by discussion with the third reviewer. Either the fixed-effects or random-effects model will be used for data synthesis based on the heterogeneity test. Either the fixed-effects or random-effects model will be used for data synthesis based on the heterogeneity test. The analysis will be conducted by RevMan 5.3 software according to Cochrane Handbook.

**Results:**

The aim of this systematic review is to provide high-quality evidence to assess the efficacy and safety of abdominal acupuncture for patients in Parkinson’s disease. The efficacy and safety of abdominal acupuncture for PD will be comprehensively assessed from the outcomes, including the effectiveness rate. The Unified Parkinson Disease Rating Scale (UPDRS) and Webster scale, Motor symptom scores utilizing UPDRS III scale, Dopamine (DA) content, and Nonmotor symptom scores employing UPDRS I scale, Activities of daily living using UDPRS II; Complications of treatment applying UPDRS IV, antioxidant ability: super oxide dismutase activity and Lipide Peroxide (LPO) content, Content of inflammatory cytokines, tumor necrosis factor-α and interleukin-1β, and adverse events as the secondary outcome.

**Conclusion:**

This systematic review will explore whether abdominal acupuncture is an effective and safe intervention for patients in Parkinson’s disease.

## 1. Introduction

Parkinson’s disease (PD) is the second most common neurodegenerative disease in the world, after Alzheimer’s disease^[1]^, and PD is also the most common disabling movement disorder.^[2]^ The main pathogenesis of PD is selective loss of neurons in the substantia nigra.^[3]^ The prevalence of PD is increasing with age and affecting approximately 1% of the population above 60 years. There is no suitable cure to postpone PD progression.^[4]^ Dopamine precursors are conventional pharmacological treatments for PD.^[5]^ In addition, Levodopa (L-dopa)can extend life expectancy that had been proved.^[6]^ However, levodopa treatment can lead to dyskinesia and OFF symptoms.^[7]^ Thus, we tried to find a complementary or alternative method to treat PD for better therapeutic effect.

Acupuncture is the ancient Chinese therapy by stimulating qi and blood to keep balance in the body.^[8]^ Previous meta-analysis proved acupuncture had positive effects on both efficacy and safety in the treatment of PD.^[9]^ Benefits are mainly reflected in improvement of PD symptoms and the reversal of dopaminergic neurodegeneration in the early stages of PD.^[10]^When combined with L-dopa, acupuncture can improve therapeutic effect, reduced dosage, and alleviate adverse effects of L-dopa.^[11]^As a form of acupuncture, Abdominal acupuncture combined with Madopa can elevate therapeutic effect of Madopa and reduce adverse effects of Madopa in patients with PD.^[12]^

However, due to small sample sizes and methodological defects, reliable scientific evidence is absent to prove the therapeutic effect of abdominal acupuncture for PD, Therefore, this research intends to adopt the method of system valuation and meta-analysis of the abdominal acupuncture for PD to evaluate the efficacy and safety.

## 2. Methods

### 2.1. Study registration

This protocol will be conducted under the preferred reporting items for systematic reviews and meta-analyses protocols guidelines.^[13]^ Furthermore, the protocol has been registered on PROSPERO as CRD42022354730.(https://www.crd.york.ac.uk/PROSPERO/display_record.php?RecordID=354730).

### 2.2. Inclusion criteria for study selection

#### 2.2..1. Types of studies.

All relevant randomized controlled trials (RCTs) in English and Chinese will be included. While non-RCTs, quasi-RCTs, cohort studies, reviews, case reports, experimental studies, and expert experience, the data of the included study is missing or incomplete, and duplicate publications will be excluded to ensure the quality of this systematic review.

#### 2.2..2. Types of participants.

Participants of different age groups with Parkinson’s disease could be included in the study, regardless of nationality, race, gender, occupation, and educational background. The main intervention in this review is abdomen acupuncture. The subject of this review is to analyze the effectiveness of abdomen acupuncture. Therefore, other acupuncture treatments that apply acupuncture to conventional acupoints on the abdomen but not based on Abdomen acupuncture theory will not be included.

#### 2.2..3. Types of interventions.

This study focuses on the RCTs of Parkinson’s disease under the treatment of abdominal acupuncture. The treatment group should be treated by abdominal acupuncture combining or not combining with western medicines. The results are anticipated to aid clinicians. All trials with an assessment of the treatment mentioned above will be included, while studies of control group could only use western medicines as the sole treatment.

#### 2.2..4. Types of outcome measures.

##### 2.2.4.1. Primary outcomes.

The primary outcomes are the overall symptom scores using the effectiveness rate, The Unified Parkinson Disease Rating Scale (UPDRS) and Webster scale, Motor symptom scores utilizing UPDRS III scale, and Dopamine(DA) content.

##### 2.2.4.2. Secondary outcomes.

The secondary outcomes of this review mainly include the following aspects: nonmotor symptom scores employing UPDRS I scale, activities of daily living using UDPRS II, complications of treatment applying UPDRS IV, SOD activity and LPO content of antioxidant ability, content of inflammatory cytokines: tumor necrosis factor-α and interleukin-1β, and adverse events.

### 2.3. Data sources

Six English databases (PubMed, Web of Science, MEDLINE, EMBASE, Springer Cochrane Library, and WHO International Clinical Trials Registry Platform) and 4 Chinese databases (Wan Fang Database, Chinese Scientific Journal Database, China National Knowledge Infrastructure Database, and Chinese Biomedical Literature Database) will be searched normatively according to the rule of each database from the inception to August 20,2022.

### 2.4. Searching strategy

Electronic literature search will be performed using PubMed, Web of Science, Medline, Embase, Springer Cochrane Library, WHO International Clinical Trials Registry Platform, Wan Fang Database, Chinese Scientific Journal Database, China National Knowledge Infrastructure Database, and Chinese Biomedical Literature Database for English and Chinese articles by August 20,2022. The Search strategy for PubMed is shown in Table 1, and similar strategies will be built and applied for other electronic databases.

**Table 1:** The search strategy for PubMed.

Number	Search terms
#1	Parkinson’s disease[MeSH Terms]
#2	Parkinson disease[MeSH Terms]
#3	Parkinson disease[MeSH Terms]
#4	Parkinson’s disease[MeSH Terms]
#5	#1 or #2 or #3 or #4
#6	Parkinson disease[Title/Abstract]
#7	Parkinson’s disease[Title/Abstract]
#8	Parkinson disease[Title/Abstract]
#9	Parkinson’s disease[Title/Abstract]
#10	#6 or #7 or #8 or #9
#11	#5 or #10
#12	Abdominal acupuncture[MeSH Terms]
#13	Abdominal needle[MeSH Terms]
#14	Abdomen acupuncture[MeSH Terms]
#15	Abdomen needle[MeSH Terms]
#16	#12 or #13 or #14 or #15
#17	Abdominal acupuncture[Title/Abstract]
#18	Abdominal needle[Title/Abstract]
#19	Abdomen acupuncture[Title/Abstract]
#20	Abdomen needle[Title/Abstract]
#21	#17 or #18 or #19 or #20
#22	#16 or #21
#23	Randomized clinical trail [Title/Abstract]
#24	Randomized controlled trail[Title/Abstract]
#25	RCT[Title/Abstract]
#26	Clinical trail[Title/Abstract]
#27	Clinical trial[Publication Type]
#28	Random* [Title/Abstract]
#29	#23 or #24 or #25 or #26 or #27
#30	#22 or #29
#31	#11 and #22 and #30

This search strategy will be modified as required for other electronic databases.

### 2.5. Data collection and analysis

#### 2.5..1. Selection of studies.

**Figure 1.**
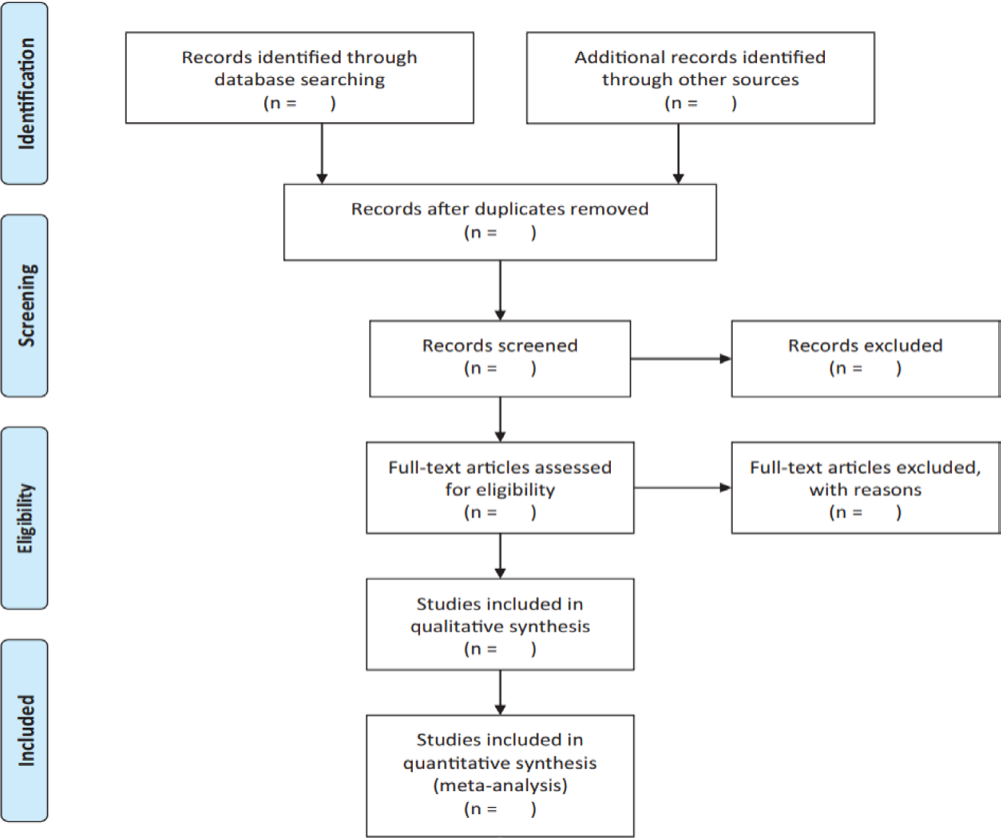
The PRISMA flow chart of the selection process.

According to the predetermined inclusion and exclusion criteria, 2 researchers will screened studies separately by title, keywords, abstracts, and full texts if needed. Each article excluded will be given a reason and recorded. Any discrepancy will be resolved through discussion or consultation a third author. During search period, EndNote X8.2 will be used for records management. The screening flow diagrams of this study will be shown in Fig. 1.

#### 2.5..2. Data extraction and management.

Two independent reviewers will separately extract the important data from the eligible study and fill in the data collection sheet. If discrepancies encountered, the decision will be made by the third reviewer. If the data needed in the records were not given, we will contact the author for more. The following information will be extracted: study details (authors, country, publication time, journal name, title, contact information), participants (inclusion and exclusion criteria, PD diagnostic criteria, age, gender, race, disease duration, baseline data), study methods (registry platform, sample size, blinding method, randomization method, allocation concealment, incomplete report or selecting report), the interventions (type of acupuncture, needles, acupoints, treatment duration, treatment frequency, practitioner, dosage of L-dopa), and the outcomes (primary and secondary outcomes shown above).

#### 2.5..3. Assessment of risk of bias.

Cochrane Handbook for Systematic Reviews will be employed to evaluate the risk of biasby 2 reviewers while the following 7 domains will be assessed: randomized sequence generation, allocation concealment, blinding of participants, personnel and outcome assessors, incomplete outcome data addressed, selective reporting, and other issue. Each potential trial of bias will be graded as high, low, and unclear. When the 2 independent reviewers failed to reach a consensus on the risk of bias assessment by negotiation, a third reviewer will make a final decision.

#### 2.5..4. Measures of treatment effect.

Data analysis will be carried out using RevMan 5.3 software. Dichotomous data will be presented as relative risks, continuous variables measuring with the same scale will be presented as with mean differences or the standardized mean differences. Effect sizes will be indicated by 95% confidence intervals (CIs).

#### 2.5..5. Unit of analysis issues.

Only the 1st experimental period data of crossover trials will be extracted in order to minimize carryover effects. For trials regarding multiple interventions, all relevant experimental groups and control groups within the trial will be combined into a single group to avoid unit-of-analysis error.

#### 2.5..6. Management of missing data.

If the data information of the included trials are found to be missing, 2 reviewers will first try to contact the original author and the corresponding authors by email. The data failed to be provided will be excluded and only the complete data will be analyzed.

#### 2.5..7. Assessment of heterogeneity.

Heterogeneity among studies is tested via chi-square test and *I*^2^ statistic tests. *P* < .05 and *I*^2^ > 50% indicated the existence of heterogeneity. Fixed-effect models are applied if there was no significant heterogeneity across studies (*I*^2^ < 50%); otherwise, random-effects models were applied.

#### 2.5..8. Assessment of reporting biases.

If more than 10 articles are included, the funnel plot of Revman 5.3 software is used to analyze publication bias. The symmetrical distribution on both sides of funnel plot data indicates that there is no publication bias, while the asymmetry on both sides may lead to publication bias. If the number of articles is less than 10, the Egger test and Begg test of Stata 14.0 software are used for statistical analysis. P < O.05 indicates the existence of publication bias, while *P* > .05 indicates the absence of publication bias.

#### 2.5..9. Data synthesis.

Data analysis and synthesis will be performed using RevMan version 5.3 software provided by the Cochrane Collaboration. The software will be used to obtain forest plots and test the heterogeneity between the included studies. Risk ratio with 95% CIs will be used for dichotomous data, while the continuous data will be analyzed by MD or standard MD with 95% CIs. Heterogeneity will be assessed by visual inspection of the forest plots and detected by standard *X*^2^ test and *I*^2^ test. When *P* > .1, *I*^2^ < 50%, it will be considered as no significant heterogeneity between the trials, and the fixed effect model will be applied for statistics, otherwise, the random effect model will be chosen. When heterogeneity occurs, sensitivity analysis or meta-regression will be performed to assess the source of heterogeneity.

#### 2.5..10. Subgroup analysis.

When heterogeneity is detected, subgroup analysis will be used. Subgroup analysis is conducted based on intervention time, gender, age and quality score of included literature, and efficiency is taken as the basis.

#### 2.5..11. Sensitivity analysis.

Sensitivity analysis is performed when there are important positive results or critical results in primary analysis. After excluding abnormal results (too large or too small samples), meta-analysis was conducted to consider whether there was any change. If there is no significant difference between the results before and after sensitivity analysis, the results are proved to be stable.

## 3. Discussion

Parkinson’s disease is the second most common neurodegenerative disease, and its prevalence has been projected to double over the next generation,^[14]^the main clinical manifestations are tremor, rigidity, and bradykinesia, affecting seriously the living standard of patients.^[15]^ Abdominal acupuncture is a special kind of acupuncture by acupuncturing the viscera and meridians in the abdomen,^[16]^ with the advantages of simple operation, convenience, and fewer side effects. Abdominal acupuncture combined with Madopa can elevate therapeutic effect of Madopa and reduce adverse effects of Madopa in patients with PD. However, the safety and efficacy of abdominal acupuncture treatment on PD remain is unclear. This study will evaluate published RCTs evidence for the efficacy and safety of EA in the treatment of PD, so as to assist clinicians on the treatments of PD and better guide clinical practice.

## Author contributions

**Conceptualization:** Yuehong Fang, Zhengzhong Liu.

**Data curation:** Sihan Dong, Zhengzhong Liu, Yilian Xu.

**Formal analysis:** Yuehong Fang, Yilian Xu, Sihan Dong.

**Funding acquisition:** Ying Su.

**Investigation:** Zhengzhong Liu.

**Methodology:** Yuehong Fang, Yilian Xu.

**Project administration:** Yilian Xu, Sihan Dong.

**Resources:** Yuehong Fang, Yilian Xu, Zhengzhong Liu.

**Software:** Yuehong Fang, Yilian Xu.

**Supervision:** Ying Su.

**Validation:** Yilian Xu, Sihan Dong.

**Visualization:** Ying Su.

**Writing—original draft:** Yuehong Fang.

**Writing—review and editing:** Ying Su.
